# Different Pediatric Acute Care Settings Influence Bronchiolitis Management: A 10-Year Retrospective Study

**DOI:** 10.3390/life13030635

**Published:** 2023-02-24

**Authors:** Carlotta Biagi, Ludovica Betti, Elisa Manieri, Arianna Dondi, Luca Pierantoni, Ramsiya Ramanathan, Daniele Zama, Monia Gennari, Marcello Lanari

**Affiliations:** 1 Pediatric Emergency Unit, IRCCS Azienda Ospedaliero-Universitaria di Bologna, 40138 Bologna, Italy; 2 Specialty School of Pediatrics, Alma Mater Studiorum, University of Bologna, 40126 Bologna, Italy; 3 Infectious Diseases Unit, Fondazione IRCCS Ca’ Granda—Ospedale Maggiore Policlinico, 20122 Milano, Italy

**Keywords:** bronchiolitis, infants, pediatric emergency department, hospitalization, short-stay observation, therapy, chest X-ray

## Abstract

Bronchiolitis is the main cause of hospitalization in infants. Diagnosis is clinical, and treatment is based on hydration and oxygen therapy. Nevertheless, unnecessary diagnostic tests and pharmacological treatments are still very common. This retrospective study aimed to evaluate whether the setting of bronchiolitis care influences diagnostic and therapeutic choices. The management of 3201 infants, referred to our Italian Tertiary Care Center for bronchiolitis between 2010 and 2020, was analyzed by comparing children discharged from the pediatric emergency department (PEDd group) undergoing short-stay observation (SSO group) and hospitalization. Antibiotic use in PEDd, SSO, and ward was 59.3% vs. 51.6% vs. 49.7%, respectively (*p* < 0.001); inhaled salbutamol was mainly administered in PEDd and during SSO (76.1% and 82.2% vs. 38.3% in ward; *p* < 0.001); the use of corticosteroids was higher during SSO and hospitalization (59.6% and 49.1% vs. 39.0% in PEDd; *p* < 0.001); inhaled adrenaline was administered mostly in hospitalized infants (53.5% vs. 2.5% in SSO and 0.2% in PEDd; *p* < 0.001); chest X-ray use in PEDd, SSO, and ward was 30.3% vs. 49.0% vs. 70.5%, respectively (*p* < 0.001). In a multivariate analysis, undergoing SSO was found to be an independent risk factor for the use of systemic corticosteroid and salbutamol; being discharged at home was found to be a risk factor for antibiotic prescription; undergoing SSO and hospitalization resulted as independent risk factors for the use of CXR. Our study highlights that different pediatric acute care settings could influence the management of bronchiolitis. Factors influencing practice may include a high turnover of PED medical staff, personal reassurance, and parental pressure.

## 1. Introduction

Acute bronchiolitis is the most frequent cause of lower respiratory tract infection (LRTI) in the first year of life [1,2,3,4] and the leading cause of hospitalization in young infants worldwide [5]. Bronchiolitis is caused by respiratory viruses that invade the epithelial cells of the small airways leading to airway inflammation and the obstruction of the lower respiratory tract [6]. Respiratory syncytial virus (RSV) is the main etiological agent, followed by rhinovirus, parainfluenza virus, human metapneumovirus, influenza virus, adenovirus, coronavirus, and human bocavirus [7,8,9]. Some infants with bronchiolitis have multiple respiratory virus co-infections [10].

Usually, symptoms of bronchiolitis begin with rhinitis or congestion and a cough, which can evolve toward symptoms of LRTI such as tachypnea, chest recession, abnormal lung sounds, such as crackles and wheezes, and eventually, decreased hydration and feeding. 

Several factors can increase the risk of severe illness including prematurity, congenital heart diseases, trisomy 21, neuromuscular disorders, immunodeficiency, and chronic lung diseases [6]. Another risk factor is being three months old or younger, probably due to the immaturity of the immunological system in younger infant and the smaller bronchial tree [5]. The most common complications are dehydration and respiratory failure [7]. Infants affected by mild bronchiolitis with no risk factors can be treated and monitored at home by parents or in the out-patient setting by primary care pediatricians. The decision to hospitalize a child with bronchiolitis presented to the emergency department must be judged on a case-by-case basis depending on clinical severity, the risk factors mentioned above, and family compliance. Admission to the short-stay observation (SSO) is warranted to strictly monitor infants for whom hospitalization is not immediately necessary [11].

International guidelines [12,13] agree that the diagnosis of bronchiolitis is clinical, and blood tests are generally not recommended in routine evaluation. Chest X-ray (CXR) should be reserved for patients admitted to the intensive care unit (ICU) or those with signs of pulmonary complications [14]. 

The treatment is supportive, based on adequate hydration and, if necessary, oxygen therapy [11,12,13]. Although routine administration of inhaled bronchodilators is not recommended, a therapeutic trial with salbutamol may be considered in infants older than six months with wheezing on auscultation. However, this therapy should be discontinued if there is no clinical improvement after the trial treatment [7,11]. Inhaled adrenaline and systemic glucocorticoids are not recommended since they are not effective in decreasing the incidence and duration of hospitalization [12,13,14]. Neither antibiotics are recommended in the treatment of bronchiolitis and their use must be avoided because of the risk of side effects, significant costs, and possible development of antimicrobial resistance. The only rational use of the antibiotics is for bacterial co-infection [14], a complication of bronchiolitis mainly represented by bacterial pneumonia, with a prevalence that varies widely in the literature, ranging from 9.7% to 42% in severe cases admitted to ICU [15,16,17].

The implementation of clinical practice guidelines has been reported to result in reductions in diagnostic and therapeutic resource use [18]; nonetheless, many children continue to undergo unnecessary tests and no evidence-based therapies [19,20]. According to this, in a previous study we have evaluated the impact of the Italian guidelines on the management of bronchiolitis in our Tertiary Care Center [20]. This study revealed that further efforts are needed to limit the prescription of ineffective therapies, particularly antibiotics.

The young age of the children affected, and the potential severity of bronchiolitis may influence practice and lead clinicians to perform CXR and prescribe no-evidence-based pharmacological therapies. The turnover of medical staff including pediatricians who have less experience in caring for infants with bronchiolitis, time pressure in PED, no possibility of a close follow-up, and parental pressure may be factors influencing clinical practice [21,22]. In this sense, the pediatric acute care setting may influence the management of infants with bronchiolitis. However, to date, the literature lacks studies assessing the possible influence of different pediatric care settings in the management of bronchiolitis. 

Thus, the purpose of this study was to evaluate the variations in diagnostic and therapeutic management of bronchiolitis comparing patients discharged from the pediatric emergency department (PEDd), patients undergoing SSO, and patients admitted to the ward. Moreover, the study aimed to evaluate whether the care setting influences diagnostic and therapeutic choices, testing it as a potential independent factor influencing pharmacological therapies and CXR use.

## 2. Materials and Methods

### 2.1. Study Design and Setting

The present was a monocentric, observational, retrospective study conducted at the Pediatric Emergency Unit of S. Orsola University Hospital in Bologna, Northern Italy. Our center is an urban, academic Tertiary Care Center, consisting of a PED with an average of 24,000 visits per year, a SSO unit, and a ward.

Patients with bronchiolitis arriving at the PED are visited in the emergency room by pediatric residents and the attending physician. They can be discharged to home, admitted to SSO unit, or to PED ward on the advice of the physician. The attending physician is a hospital pediatrician who can be part of or not of the PED staff.

The SSO unit is a 6-bed unit located adjacent to the emergency room. It can accommodate lower acuity children, who need short-term monitoring, observation, focused diagnostics, or therapeutic interventions. The length of stay in SSO unit is set between 6 and 72 h, after which the patient can be discharged or hospitalized in the PED ward. The SSO unit is under the clinical governance of the PED staff with the exception of weekends and public holidays when the attending pediatrician can be part of or not of the PED staff.

The PED ward is a 28-bed ward run by dedicated PED staff of 11 pediatricians who are experienced in caring for infants with bronchiolitis. The length of hospital stay in ward is set at more than 48 h.

All infants referred for acute bronchiolitis to our Pediatric Emergency Unit from 1 November 2010 to 31 March 2020 were included in the study.

Exclusion criteria were individuals older than 12 months of age and parents or legal guardians who refused the collection of data.

The primary endpoint of the study was to compare the use of CXR and pharmacological therapies in three different groups: infants discharged from the PED (PEDd), infants undergoing SSO, and hospitalized infants.

Secondary endpoints were to identify predictors of the type of care settings and predictors of the use of CXR and pharmacological therapies in children with bronchiolitis, with particular regard to the type of care setting. 

### 2.2. Data Collection

Files of children discharged from the Pediatric Emergency Unit with a diagnosis of bronchiolitis (diagnosis codes 466.1 and 466.19 according to the 9th version of the ICD) were retrospectively reviewed. We included the following information for each patient: demographic data (age in months and sex), risk factors for severe bronchiolitis (e.g., previous episodes of apnea, chronic pulmonary disease, congenital heart disease, immunodeficiency, severe neurological or muscle disease, and prematurity), any previous PED access for the same episode, number of days since symptoms onset, vital signs at arrival at the hospital, bronchiolitis severity according to the Italian inter-society consensus document (Table S1) [11], any eating difficulties, laboratory and radiological investigations (blood count, C-reactive protein (CRP), RSV test, and CXR), discharge modality (home, SSO, or admission to the ward), bronchopneumonia and other complications, and data on therapies (intravenous hydration, supplemental oxygen, antibiotics, steroids, nebulized adrenaline, or salbutamol).

### 2.3. Statistical Analysis

Categorical variables are expressed as numbers and percentages and they were compared by Pearson Chi-square test or Fisher’s exact test, as appropriate. After a Bartlett test for homoskedasticity, continuous variables are reported as median and relative interquartile range (IQR) or reported as mean and standard deviation (SD) as appropriate, and they were, respectively, compared by the Kruskal–Wallis test or by the one-way ANOVA. *p* < 0.05 denotes statistical significance.

The Kruskal–Wallis test and the one-way ANOVA were followed by post hoc comparisons, respectively, with the Dunn Test and the Tukey Test to compare the subgroups on continuous variables; statistically significant differences in the subgroups are reported, highlighting the relationship between the groups. The standardized residuals were used in the post hoc comparison to compare categorical variables among groups; the groups that significantly contributed to the rejection of the independence hypothesis are reported with the symbol “−”, which indicates that the variable has a lower frequency in that group than it might be expected by chance and with the symbol “+”, which indicates that the variable has a higher frequency in that group than it might be expected by chance. 

After having performed bivariate tests, we carried out a multinomial logistic regression to evaluate the influence of different factors on the different care setting of a child with acute bronchiolitis. The factors that were included in the model were those that were significant at a 0.2 level in the bivariate tests. The potential factors tested were as follows: age groups (≤3 months, ≤6 months, and >6 months), prematurity, other risk factors for severe bronchiolitis, feeding difficulties at admission, number of days between the onset of symptoms and hospital access, SpO2 value, RR, clinical severity, RSV, fever, and previous PED access for the same episode of bronchiolitis.

Bivariate analyses were conducted to evaluate independent factors, which can influence pharmacological therapies (antibiotics, corticosteroids, inhaled salbutamol, and adrenaline) and CXR use, including the pediatric acute care settings (PEDd, SSO unit, and ward). Variables tested as potential independent risk factors were as follows: acute care settings, age groups (≤3 months, ≤6 months, and >6 months), prematurity, other risk factors for severe bronchiolitis, clinical severity, fever, bronchopneumonia, and CXR. Since inhaled adrenaline was almost exclusively administered in ward, the bivariate tests and the following multivariate analysis for this therapy were created only for hospitalized patients. Moreover, when analyzing the independent factors for the use of CXR, the variables bronchopneumonia and CXR were not included in the analyses. For each therapy analyzed, independent variables that were significant at a 0.2 level in the bivariate tests were included in a multivariate binary logistic regression model. Results of the multivariate analyses are reported as odds ratio (OR) values and relative 95% confidence intervals (95% CI).

All data analyses were performed using R, version 4.0.3, and RStudio, version 1.3.1093, for Windows.

### 2.4. Ethical Statement

This retrospective chart review study was in accordance with the 1964 Helsinki Declaration and its later amendments. The study was approved by the local Ethical Committee (protocol number 1062/2020/Oss/AOUbo).

## 3. Results

During the study period, a total of 3201 infants aged 0–1 years was admitted to our PED for acute bronchiolitis. A total of 1925 (60.1%) were males and the median age was 6.1 months. Of all the children, 1640 (51.2%) were discharged after having been assessed in the PED, 314 (9.8%) were admitted to SSO, and 1247 (39%) were hospitalized in our pediatric unit ward. 

The main features of the three study groups are described in Table 1.

Regarding the evidence-based supportive treatment, intravenous hydration and oxygen therapy were administered more often in hospitalized infants (82.2% in the hospitalized group vs. 11.5% in the SSO group vs. 0.2% in the PEDd group, *p* < 0.001 for intravenous hydration and 42.7% in the hospitalized group vs. 7.3% in the SSO group and 0.1% in the PEDd group, *p* < 0.001 for oxygen therapy).

### 3.1. Primary Endpoint

The use of pharmacological treatments and CXR in the three study groups are shown in Table 2. 

The administration of systemic corticosteroids in PEDd, SSO, and the ward was 39.0% vs. 59.6% vs. 49.1%, respectively (*p* < 0.001). Antibiotic use was higher than expected by chance in children discharged from PED (59.3%), while it was lower than expected by chance in hospitalized patients (49.7%) (*p* < 0.001). Nebulized adrenaline administration in PEDd, SSO, and the ward was 0.2% vs. 2.5% vs. 53.5%, respectively (*p* < 0.001). Inhaled salbutamol was mainly administered in the PEDd and SSO groups (76.1% and 82.2%, respectively, vs. 38.3% in hospitalized group, *p* < 0.001).

The use of CXR was higher than expected by chance in hospitalized children and lower than expected by chance in PEDd (70.5% and 30.3%, *p* < 0.001).

### 3.2. Secondary Endpoints

We performed a multivariate analysis to evaluate the weight of predisposing factors for the different care setting of a child with acute bronchiolitis.

The comparison of children undergoing SSO to those discharged from PED revealed that being under 6 months of age (*p* = 0.010), prematurity (*p* < 0.001), other known risk factors (*p* = 0.005), and feeding difficulties at admission (*p* = 0.009) were statistically significant predictors for undergoing SSO. Additionally, the severity of bronchiolitis (*p* < 0.001), the presence of fever (*p* = 0.001), and a previous PED access for bronchiolitis (*p* = 0.001) were statistically significant risk factors for the management in SSO. On the contrary, the higher SpO2 value (*p* = 0.001) and the higher number of days since symptoms onset (*p* = 0.018) appeared to be protective factors against SSO management. 

The comparison between hospitalized infants and those discharged from PED showed that both the age groups, ≤3 months and ≤6 months, were associated with hospitalization (*p* < 0.001). Other risk factors for hospitalization were as follows: prematurity and other known risk factors for severe bronchiolitis, previous PED access for the same bronchiolitis episode, fever, feeding difficulties, and the severity of bronchiolitis at admission (*p* < 0.001). The higher SpO2 value appeared to be a protective factor against hospitalization (*p* < 0.001).

Details of OR and relatives 95% CI for multivariate analysis are reported in Table S2.

### 3.3. Pharmacological Therapies and CXR Predictors

After having performed appropriate bivariate tests (Table S3), we carried out multivariate analyses to identify the predictors of pharmacological therapies and CXR use among patients with bronchiolitis. The data are reported in Table 3.

Of all the children included in the study, 1438 (44.9%) received systemic corticosteroid, 639 (44.4%) of whom were discharged from PED, 187 (13%) underwent SSO, and 612 (42.6%) were hospitalized. 

In the multivariate analysis, undergoing SSO compared to PEDd was a risk factor for the use of systemic corticosteroids (OR = 1.967 and *p* < 0.001), together with the presence of bronchopneumonia (OR = 1.549 and *p* < 0.001), and the presence of moderate or severe bronchiolitis compared to mild disease (respectively, OR = 3.267 and *p* < 0.001; OR = 6.065 and *p* < 0.001). On the contrary, the presence of fever (OR = 0.527 and *p* < 0.001) and being 3 months old or under compared with being aged > 6 months (OR = 0.643 and *p* < 0.001) resulted as protective factors.

Regarding antimicrobial therapy, 1755 (54.8%) of the children included in the study received antibiotics, most of whom were discharged from PED (*n* = 973; 55.4%). Severe bronchiolitis compared to mild disease (OR = 2.365 and *p* < 0.035), together with fever (OR = 2.870 and *p* < 0.001), bronchopneumonia (OR = 7.655 and *p* < 0.001), and the use of CXR (OR = 1.933 and *p* < 0.001) resulted as risk factors for the use of antibiotics. Undergoing SSO (OR = 0.514 and *p* < 0.001) and hospitalization (OR = 0.415 and *p* < 0.001) compared to PEDd resulted as protective risk factors for antibiotics, together with the being under 3 months of age (OR = 0.552 and *p* < 0.001) and under 6 months (OR = 0.681 and *p* < 0.001), in comparison with being aged > 6 months. 

Of all the children included in this study, 679 (21.2%) received nebulized adrenaline, 667 (98.2%) of which were during hospitalization. Since almost all the administrations concerned hospitalization, the bivariate model and the multivariate analysis were created only for hospitalized patients (*n* = 1239). Risk factors for the use of adrenaline aerosols were as follows: aged under 3 and 6 months compared to aged > 6 months (respectively, OR = 5.623 and *p* < 0.001; OR = 3.353 and *p* < 0.001), moderate (OR = 2.319 and *p* < 0.001) and severe bronchiolitis (OR = 4.909 and *p* < 0.001) compared to mild disease, the use of CXR (OR = 2.552 and *p* < 0.001), and the presence of bronchopneumonia (OR = 1.889 and *p* < 0.001).

Regarding inhaled salbutamol, 1983 (61.9%) children received the therapy, mostly infants who were older than six months (80% vs. 45%). Undergoing SSO (OR = 1.492 and *p* = 0.020) compared to PEDd, moderate bronchiolitis (OR = 1.599 and *p* < 0.001) compared to mild disease, and the presence of bronchopneumonia (OR = 1.483 and *p* = 0.002) were risk factors for the use of salbutamol aerosols. On the contrary, hospitalization was a protective factor for the use of this therapy compared to PEDd (OR = 0.331 and *p* < 0.001), together with being aged under 3 months and 6 months (respectively, OR = 0.097 and *p* < 0.001; OR = 0.662 and *p* < 0.001). 

Of all the children included in this study, 1530 (47.8%) underwent CXR. In the multivariate analysis, SSO (OR = 2.128 and *p* = 0.001) and hospitalization (OR = 5.679 and *p* < 0.001) compared to PEDd, together with prematurity (OR = 1.508 and *p* = 0.015), and the presence of fever (OR = 14.83 and *p* = 0.001) resulted as risk factors for the use of CXR.

## 4. Discussion

The present study reported the variations in the therapeutic and diagnostic choices between patients discharged from the PED, patients undergoing SSO, and patients admitted to the ward. Moreover, it highlights that the setting in which bronchiolitis is managed (PEDd, SSO, and ward) could influence the management of the disease. Undergoing SSO was found to be an independent risk factor for the use of systemic corticosteroid and salbutamol; being discharged at home was found to be a risk factor for antibiotic prescription; undergoing SSO and hospitalization resulted as independent risk factors for the use of CXR.

The majority of our patients were males (60%) with a median age of six months, in line with previous studies [1,23,24]. As expected, hospitalized patients were significantly younger compared to PEDd and SSO, and they more frequently had one or more risk factors for the development of severe bronchiolitis, and presented feeding difficulties and a more severe disease. This is in line with the data from the literature [1]. RSV positivity was 64.4% and resulted significantly more in the hospitalized patients. This percentage is coherent with the literature, ranging from 41% to 83% for those patients [24]. It should be noted that viral testing is not routinely recommended as it does not change the clinical management of bronchiolitis, and it is more frequently performed on hospitalized infants to aid in patient cohorting during an epidemic [2]. According to this, only 1502 (46.9%) patients from our study population were tested for RSV, of which 1232 (82%) were hospitalized, and this may have affected our results. 

As expected, moderate and severe bronchiolitis, together with the presence of complications, mostly represented by bronchopneumonia, were more frequent in hospitalized patients.

Intravenous hydration was administered more often in the hospitalized group, according to the higher number of patients with feeding difficulties. Additionally, oxygen therapy was mostly used in hospitalized patients, which is in line with the clinical picture severity characterized by a higher percentage of moderate and severe forms of bronchiolitis with respiratory distress and hypoxia.

The predictors of the type of acute care setting (PEDd vs. SSO vs. hospitalization) for bronchiolitis that emerged in our study are essentially in line with the indications of the Italian guidelines [11]. As expected, the main predictors of hospitalization and SSO were a younger age, prematurity, and other pre-existing risk factors (e.g., previous episodes of apnea, chronic pulmonary disease, congenital heart disease, immunodeficiency, severe neurological or muscle disease, and prematurity), feeding difficulties, low SpO_2_, and severe bronchiolitis, according to the Italian inter-society consensus document [11]. Moreover, the presence of fever and a previous PED access for the same episode of bronchiolitis resulted as independent risk factors for both hospitalization and SSO. Fever by itself is not a sign of bronchiolitis severity; however, clinicians may have a concern about missing an alternative diagnosis, mainly a bacterial infection, and this may lead to admitting the child to SSO or a hospital ward. Similarly, a previous PED access for the same episode may reflect poor parental compliance in managing the child at home, which may lead clinicians to SSO or hospital admission. Finally, in our study, higher number of days of symptoms since the onset of bronchiolitis resulted as a protective factor for SSO; this is probably because, if the symptoms have already been present for several days, clinicians may assume that the acute phase of the disease has passed, and that bronchiolitis may be adequately managed in the outpatient setting by parents and primary care pediatricians.

While the choice of the care setting for patients with bronchiolitis mainly reflect the recommendations, our study highlighted that it is far more difficult to observe the guidelines concerning therapeutic and diagnostic management.

Regarding the administration of nebulized salbutamol in our population study, it concerned around 62% of patients, mainly PEDd and SSO patients. This high percentage may be explained by the fact that until 2014, nebulized salbutamol was widely used in bronchiolitis patients with a prevalence of wheezing to chest auscultation. Moreover, the Italian inter-society consensus document published in 2014 still allows a therapeutic attempt in patients with bronchiolitis older than 6 months, in particular in those with a family history of allergy, asthma and/or atopy [11]. According to this, we reported a higher rate of salbutamol use in patients aged 6–12 months compared to younger children. As a matter of fact, the PEDd and SSO groups were mainly accounted for by patients who were older than six months. Moreover, the high turnover of medical staff in PED and SSO including pediatricians with less experience, together with time pressure and parental pressure may be factors influencing clinical practice [21]. According to this, in our study, hospitalization resulted as a protective factor in the multivariate analysis for the use of inhaled salbutamol, together with being aged under 6 months. 

Nebulized adrenaline was administered in 21% of patients, and most of them hospitalized (98.2%). The risk factors for the use of adrenaline were being aged under 3 and 6 months, moderate and severe bronchiolitis, bronchopneumonia, and the use of CXR. Considering that salbutamol is not recommended for children under 6 months of age, physicians may feel compelled to prescribe adrenaline in young infants with moderate or severe bronchiolitis, either for personal reassurance or parental pressure [22]. 

Systemic corticosteroids were administered in 45% of the study population, which is significantly more in the SSO and hospitalized group compared to PEDd. This percentage is higher than the one reported in the literature, where the use of corticosteroids is estimated in about 10–25% of patients with bronchiolitis [25,26]. The main reasons for this wrong practice could be the anxiety of the clinicians to treat a potentially severe condition and, sometimes, parental pressure [22]. In support of this, in our multivariate analysis, the presence of moderate and severe bronchiolitis together with bronchopneumonia were risk factors for the administration of corticosteroids. Moreover, undergoing SSO was also found to be an independent risk factor for the prescription of systemic corticosteroids. 

The literature findings regarding the percentage of antibiotic use in bronchiolitis are very discordant, ranging from 3.5% up to 53%, with considerable differences between the various countries [27,28]. In Italy, Barbieri et al. [29] reported a high rate of antibiotic use by primary care pediatricians (37% of cases). Our study confirmed the high consumption of antibiotics in Italy, documenting an overall use of antibiotic in 55% of the study population. As expected, in line with the literature, the presence of fever—which may indicate a secondary bacterial infection—and bronchopneumonia resulted as risk factors for the use of antibiotic. Additionally, children aged ≤ 6 months and under were associated with a higher use of antibiotic, which can be explained by the fear of missing a bacterial co-infection in young infants, even if invasive bacterial infections in these patients are rare, with a risk of bacteremia or meningitis at less than 1% [24]. 

In our study, undergoing SSO and hospitalization resulted as independent protective factors for the prescription of antibiotic therapy, which are mostly prescribed in PEDd. This result may firstly depend on the concern for missing an alternative diagnosis, such as pneumonia, in infants discharged from PED with no possibility of a close follow-up. Secondly, the pressure of parents who want antibiotics to be prescribed, or, if already started, to be continued, may influence clinicians [21]. Moreover, some clinicians with a lack of knowledge and limited experience in the management of bronchiolitis could perform a CXR to rule out pneumonia and subsequently prescribe antibiotics [21]. Indeed, from our data, another risk factor for antibiotics was the use of CXR. It has been previously reported that the probability of prescribing antibiotics is higher when CXR is performed, regardless of the actual presence of bronchopneumonia. This is due to the similar radiographic appearance of infiltrate and atelectasis [30,31], confirming that CXR is not an adequate technique for these patients [30,32] 

According to the guidelines, CXR should be considered only in patients with a presentation that is not classic for bronchiolitis, and for severe cases with a late worsening illness, or with signs of pneumonia, such as persistently focal crackles and fever [24]. Nevertheless, it continues to be performed frequently, in up to 60–70% of PED visits [27,28,33,34] and in about 50% of hospitalized patients with bronchiolitis [16,35]. According to this, in our study, 47.8% of the patients underwent CXR, mainly among hospitalized children. In the multivariate analysis carried out to identify the predictors of CXR use, we found that undergoing SSO and hospitalization, together with the presence of fever or prematurity at birth resulted as the independent risk factors for the use of CXR. The clinical severity of bronchiolitis was unexpectedly not associated with the use of CXR, suggesting that clinicians mainly prescribe radiography to allay fears of missing a bacterial LRTI. In this sense, a lung ultrasound, which is not associated with a risk of ionizing radiation like CXR, may represent a value supplemental tool in the diagnostic work-up of bronchiolitis [16,36]. Another strategy aiming to reduce CXR and antibiotic prescription in PED may be the use of point-of-care biomarkers (e.g., C-reactive protein or procalcitonin) to rule out bacterial co-infections [37].

Our study presents some limitations. First, this is a monocentric study, thus further multicenter analyses are needed to confirm our results. Second, the unequal sample sizes could have slightly affected the power of the tests and the confidence intervals. Third, given its retrospective design, causality cannot be ascertained, and factors influencing practice (e.g., parental pressure and high turnover in medical staff) cannot be explored because the retrospective design of the study precluded the collection of these data. Moreover, in our work, some variables were not considered, such as social and environmental factors (e.g., distance from home to hospital, humid or cold dwelling, and family crowding) that may have influenced the acute care setting choices. Finally, the publication of the first Italian inter-society consensus document in 2014 could have had an impact on the results, since the study period is from 2010 to 2020. However, the main international guidelines, such as the guidelines issued by the American Academy of Pediatrics in 2006 [14], have already reported that the cornerstone of bronchiolitis management is represented by supportive therapy (oxygen and fluid supplementation), and there is no evidence of efficacy for numerous therapies that are commonly used when treating bronchiolitis (bronchodilators, steroids, and antibiotics). 

Strength points of the study are the long observation period and the large sample. 

## 5. Conclusions

Our study highlights that the management of bronchiolitis varies between PEDd patients, children undergoing SSO, and hospitalized patients, and that the pediatric acute care setting could influence diagnostics and therapeutic choices. Undergoing SSO was found to be an independent risk factor for systemic steroid and salbutamol use, while being discharged at home showed up as a risk factor for an antibiotic prescription. Finally, undergoing SSO and hospitalization resulted as independent risk factors for the use of CXR.

According to the literature, many factors may influence practice, including high turnover of medical staff in PED and SSO, personal reassurance, and parental pressure. Further efforts are needed to standardize bronchiolitis diagnosis and treatment and limit unnecessary prescriptions. Possible strategies include the development of specific resident guidelines to improve physicians’ education and the creation of family-friendly material to avoid parental pressure. The use of CXR should likewise be restricted. Lung ultrasound examinations and point-of-care biomarkers in PED may be valuable tools to rule out bacterial co-infections and reduce CXR and antibiotic prescriptions. Further studies are warranted to better evaluate their utility in the clinical management of infants with bronchiolitis. 

## Figures and Tables

**Table 1:** Demographic and clinical data of the study population.

	(A) PEDd (*n* = 1640)	(B) SSO (*n* = 314)	(C) Hospitalized (*n* = 1247)	*p*-Value (Test)	Post Hoc Comparison
Sex, *n* (%), male	1019 (62.1)	191 (60.8)	715 (57.3)	0.032 (Chi)	A+, C−
Age (months), median (IQR)	7.40 (5.13)	7.18 (5.79)	3.13 (3.68)	**<0.001** (K)	A, B > C
Age class, *n* (%):				**<0.001** (Chi)	
≤3 months	146 (8.9)	30 (9.6)	609 (48.8)		A−, B−, C+
3–6 months	427 (26)	94 (29.9)	377 (30.2)		A−, C+
≥6 months	1067 (65.1)	190 (60.5)	261 (20.9)		A+, B+, C−
Prematurity, *n* (%)	24 (1.5)	13 (4.1)	201 (16.1)	**<0.001** (Chi)	A−, B−, C+
Other risk factors, *n* (%)	28 (1.7)	15 (4.8)	258 (20.7)	**<0.001** (Chi)	A−, B−, C+
Feeding difficulties, *n* (%)	374 (22.8)	122 (38.9)	853 (68.4)	**<0.001** (Chi)	A−, C+
Days of symptoms, median (IQR)	3 (4)	3 (2)	3 (3)	**<0.001** (K)	A, C > B
Previous access, *n* (%)	128 (7.8)	46 (14.6)	405 (32.5)	**<0.001** (Chi)	A−, C+
Fever, *n* (%)	175 (10.8)	53 (16.9)	152 (12.3)	**0.009** (Chi)	A−, B+
RR, mean (SD)	43.87 ± 10.57	50.04 ± 11.62	53.55 ± 10.87	**<0.001** (A)	C > B > A
SpO_2_, median (IQR)	98 (2)	97 (4)	97 (3)	**<0.001** (K)	A > B > C
Clinical severity °, *n* (%)				**<0.001** (F)	
Mild	1550 (94.5)	248 (79)	719 (57.7)		A+, C−
Moderate	90 (5.5)	62 (19.7)	488 (39.1)		A−, C+
Severe	0	4 (1.3)	40 (3.2)		A−, C+
RSV (*n* = 1502), *n* (%)	85 (51.5)	70 (67.3)	812 (65.9)	**0.001** (Chi)	A−, C+
Bronchopneumonia, *n* (%)	105 (6.4)	50 (15.9)	371 (29.8)	**<0.001** (Chi)	A−, C+
Other complications, *n* (%)	0 (0)	0 (0)	14 (1.1)	**<0.001** (F)	A−, C +

*p*-values in bold are statistically significant and refer to any differences between the groups. Significant post hoc comparisons of scale variables are reported with the symbol “>”, which indicates in which group the variable has the highest value. Significant post hoc comparisons of categorical variables are reported with the symbol “−” or with the symbol “+”, which indicate that in the related group observed frequencies were, respectively, lower or higher than those expected by chance according to the Italian guidelines on bronchiolitis [11]. A: one-way ANOVA; Chi: Chi-square test; F: Fisher’s exact test; IQR: interquartile range; K: Kruskal–Wallis test; PEDd: discharged from pediatric emergency department; RR: respiratory rate; RSV: respiratory syncytial virus; SD: standard deviation; SpO_2_: peripheral oxygen saturation; and SSO: short-stay observation; ° according to the Italian guidelines [11].

**Table 2:** Use of pharmacological treatments and CXR in the three groups of patients.

	(A) PEDd (*n* = 1640)	(B) SSO (*n* = 314)	(C) Hospitalized (*n* = 1247)	*p*-Value (Test)	Post Hoc Comparison
Systemic corticosteroids, *n* (%)	639 (39.0)	187 (59.6)	612 (49.1)	**<0.001** (Chi)	A−, B+, C+
Antibiotic, *n* (%)	973 (59.3)	162 (51.6)	620 (49.7)	**<0.001** (Chi)	A+, C−
Inhaled adrenaline, *n* (%)	4 (0.2)	8 (2.5)	667 (53.5)	**<0.001** (Chi)	A−, B−, C+
Inhaled salbutamol, *n* (%)	1248 (76.1)	258 (82.2)	477 (38.3)	**<0.001** (Chi)	A+, B+, C−
CXR, *n* (%)	497 (30.3)	154 (49)	879 (70.5)	**<0.001** (Chi)	A−, C+

*p*-values in bold are statistically significant and refer to any differences between the groups. Significant post hoc comparisons are reported with the symbol “−” or with the symbol “+”, which indicate that in the related group observed frequencies were, respectively, lower or higher than those expected by chance. Chi: Chi-square test; CXR: chest X-ray; PEDd: discharged from pediatric emergency department; and SSO: short-stay observation.

**Table 3:** Multivariable logistic regressions on determinants of the administration of pharmacological therapies and of CXR use. Dependent variables are indicated in bold in the first column.

	OR	95% CI	*p*-Value
**Systemic corticosteroids**			
SSO	1.967	1.520–2.552	**<0.001**
Hospitalization	0.975	0.782–1.215	0.821
Age ≤ 3 months	0.643	0.517–0.798	**<0.001**
Age ≤ 6 months	0.915	0.764–1.096	0.337
Prematurity	1.137	0.836–1.547	0.415
Other risk factors	1.171	0.886–1.547	0.267
Severity moderate °	3.267	2.659–4.026	**<0.001**
Severity severe °	6.065	2.945–13.789	**<0.001**
Fever	0.527	0.413–0.668	**<0.001**
CXR	1.092	0.923–1.291	0.303
Bronchopneumonia	1.967	1.242–1.934	**<0.001**
**Antibiotics**			
SSO	0.514	0.392–0.673	**<0.001**
Hospitalization	0.415	0.331–0.519	**<0.001**
Age ≤ 3 months	0.552	0.441–0.690	**<0.001**
Age ≤ 6 months	0.681	0.565–0.821	**<0.001**
Severity moderate °	0.841	0.676–1.045	0.119
Severity severe °	2.365	1.089–5.478	**0.035**
Fever	2.870	2.201–3.774	**<0.001**
CXR	1.933	1.623–2.305	**<0.001**
Bronchopneumonia	7.655	5.770–10.280	**<0.001**
**Adrenaline aerosols**			
Age ≤ 3 months	5.623	3.963–8.056	**<0.001**
Age ≤ 6 months	3.353	2.336–4.849	**<0.001**
Other risk factors	0.908	0.665–1.242	0.547
Severity moderate °	2.319	1.789–3.019	**<0.001**
Severity severe °	4.909	2.207–12.153	**<0.001**
Fever	0.730	0.495–1.075	0.111
CXR	2.552	1.926–3.393	**<0.001**
Bronchopneumonia	1.889	1.409–2.545	**<0.001**
**Salbutamol aerosols**			
SSO	1.492	1.073–2.105	**0.020**
Hospitalization	0.331	0.260–0.420	**<0.001**
Age ≤ 3 months	0.097	0.076–0.122	**<0.001**
Age ≤ 6 months	0.662	0.542–0.810	**<0.001**
Prematurity	0.962	0.688–1.346	0.821
Other risk factors	1.011	0.744–1.376	0.944
Severity moderate °	1.599	1.261–2.033	**<0.001**
Severity severe °	1.357	0.670–2.766	0.397
CXR	0.850	0.700–1.033	0.101
Bronchopneumonia	1.483	1.152–1.915	**0.002**
**CXR**			
SSO	2.128	1.657–2.732	**<0.001**
Hospitalization	5.679	4.577–7.071	**<0.001**
Age ≤ 3 months	0.800	0.637–1.001	0.052
Age ≤ 6 months	0.915	0.758–1.105	0.358
Prematurity	1.508	1.088–2.111	**0.015**
Other risk factors	0.983	0.736–1.317	0.907
Severity moderate °	0.997	0.809–1.228	0.976
Severity severe °	2.179	1.002–5.459	0.068
Fever	1.483	1.171–1.880	**0.001**

*p*-values in bold are statistically significant. CI: confidence interval; OR: odds ratio; and SSO: short-stay observation unit, ° according to the Italian guidelines [11].

## Data Availability

The data presented in this study are available on request from the corresponding author. The data are not publicly available due to reasons concerning privacy.

## Supplementary Materials

The following supporting information can be downloaded at https://www.mdpi.com/article/10.3390/life13030635/s1. Table S1: Severity index of bronchiolitis. Table S2: Multivariate analysis of the predictors of the care setting for patients with bronchiolitis. Table S3: Bivariate analysis of the predictors of no-evidence-based therapies and CXR use.
