# Supplementary material for: Different Pediatric Acute Care Settings Influence Bronchiolitis Management: A 10-Year Retrospective Study
Source: Life (Basel). 2023 Feb 24;13(3):635. doi: 10.3390/life13030635 (PMC10056632; doi:10.3390/life13030635)
Supplement: Supplementary file 1 [file life-13-00635-s001.zip › life-2116340-supplementary.pdf]

**Table S1.** Severity index of bronchiolitis [11].

| | Mild | Moderate | Severe |
|---|---|---|---|
| **Respiratory rate** | Normal to slightly increased | Increased | Markedly increased compared to normal values |
| **Respiratory effort** | Mild chest wall retraction | Tracheal tug | Marked chest wall retraction |
| | | Nasal Flare | Nasal Flare |
| | | Moderate chest wall retraction | Grunting |
| **Oxygen Saturation** | No supplemental oxygen requirement, O2 saturations > 95% | Saturation 90-95% | Saturations < 90%, may not be corrected by O2 |
| **Feeding** | Normal to slightly increased | 50-75% of normal feeds | < 50% of feeds, unable to feed |
| **Apnoea** | Nil | May have brief episodes | May have increasing episodes |

**Table S2.** Multivariate analysis of the predictors of the care setting for patients with bronchiolitis.

| Care setting | Predictors | OR | 95% CI | *p*-value |
|---|---|---|---|---|
| **SSO vs PEDd** | Age ≤ 3 months | 1.483 | 0.958-2.295 | 0.077 |
| | Age ≤ 6 months | 1.459 | 1.094-1.946 | **0.010** |
| | Prematurity | 2.626 | 1.266-5.446 | **0.009** |
| | Other risk factors* | 2.589 | 1.325-5.058 | **0.005** |
| | Feeding difficulties | 1.863 | 1.431-2.425 | **< 0.001** |
| | Days of symptoms | 0.966 | 0.938-0.994 | **0.018** |
| | $SpO_2$ | 0.889 | 0.828-0.955 | **0.001** |
| | Clinical severity° | 3.174 | 2.085-4.834 | **< 0.001** |
| | Fever | 1.779 | 1.252-2.529 | **0.001** |
| | Previous PED access | 1.855 | 1.276-2.699 | **0.001** |
| **Hospitalized vs PEDd** | Age ≤ 3 months | 37.368 | 27.232-51.277 | **< 0.001** |
| | Age ≤ 6 months | 5.677 | 4.302 -7.490 | **< 0.001** |
| | Prematurity | 7.945 | 4.469-14.122 | **< 0.001** |
| | Other risk factors* | 13.073 | 7.787-21.946 | **< 0.001** |
| | Feeding difficulties | 4.879 | 3.886 - 6.127 | **< 0.001** |
| | Days of symptoms | 1.010 | 0.992- 1.029 | 0.263 |
| | $SpO_2$ | 0.787 | 0.739-0.837 | **< 0.001** |
| | Clinical severity° | 6.588 | 4.570- 9.497 | **< 0.001** |
| | Fever | 2.226 | 1.570 -3.156 | **< 0.001** |
| | Previous PED access | 4.287 | 3.174- 5.791 | **< 0.001** |

Significant p-values are indicated in bold. CI: confidence interval; OR: odds ratio; PEDd: discharged from the Pediatric Emergency Department; SpO2: peripheral oxygen saturation; SSO: Short-stay Observation unit; *Other risk factors include: previous episodes of apnea, history of chronic lung or congenital heart disease, immunodeficiency, severe neurological or muscular disease; ° according to the Italian inter-society consensus document on bronchiolitis [11].

**Table S3.** Bivariate tests of the predictors of pharmacological therapies and CXR use.

| | Not administered therapy / Not performed CXR | Administered therapy / Performed CXR | *p*-value |
|---|---|---|---|
| **Systemic corticosteroids, n (%)** | **1763 (55.1%)** | **1438 (44.9%)** | |
| PEDd | 1001 (56.8%) | 639 (44.4%) | **< 0.001** |
| SSO | 127 (7.2%) | 187 (13%) | **< 0.001** |
| Hospitalization | 635 (36%) | 612 (42.6%) | **< 0.001** |
| Age ≤ 3 months | 464 (26.3%) | 321 (22.3%) | **0.033** |
| Age ≤ 6 months | 482 (27.3%) | 416 (28.9%) | **0.033** |
| Age > 6 months | 817 (46.3%) | 701 (48.7%) | **0.033** |
| Prematurity | 106 (6%) | 132 (9.2%) | **< 0.001** |
| Other risk factors* | 134 (7.6%) | 167 (11.6%) | **< 0.001** |
| Severity_mild° | 1544 (87.6%) | 973 (67.7%) | **< 0.001** |
| Severity_moderate° | 210 (11.9%) | 430 (29.9%) | **< 0.001** |
| Severity_severe° | 9 (0.5%) | 35 (2.4%) | **< 0.001** |
| Fever | 241 (13.8%) | 139 (9.8%) | **< 0.001** |
| CXR | 781 (44.3%) | 749 (52.1%) | **< 0.001** |
| Bronchopneumonia | 218 (12.4%) | 308 (21.4%) | **< 0.001** |
| **Antibiotics** | **1446 (45.2%)** | **1755 (54.8%)** | |
| PEDd | 667 (46.1%) | 973 (55.4%) | **< 0.001** |
| SSO | 152 (10.5%) | 162 (9.2%) | **< 0.001** |
| Hospitalization | 627 (43.4%) | 620 (35.3%) | **< 0.001** |
| Age ≤ 3 months | 443 (30.6%) | 342 (19.5%) | **< 0.001** |
| Age ≤ 6 months | 439 (30.4%) | 459 (26.2%) | **< 0.001** |
| Age > 6 months | 564 (39%) | 954 (54.4%) | **< 0.001** |
| Prematurity | 113 (7.8%) | 125 (7.1%) | 0.500 |
| Other risk factors* | 139 (9.6%) | 162 (9.2%) | 0.758 |
| Severity_mild° | 1125 (77.8%) | 1392 (79.3%) | **0.002** |
| Severity_moderate° | 311 (21.5%) | 329 (18.7%) | **0.002** |
| Severity_severe° | 10 (0.7%) | 34 (1.9%) | **0.002** |
| Fever | 87 (6.1%) | 293 (16.8%) | **< 0.001** |
| CXR | 552 (38.2%) | 978 (55.7%) | **< 0.001** |
| Bronchopneumonia | 72 (5%) | 454 (25.9%) | **< 0.001** |
| **Adrenaline aerosols, n (%)** | **580 (46.5%)** | **667 (53.5%)** | |
| Age ≤ 3 months | 228 (39.3%) | 381 (57.1%) | **< 0.001** |
| Age ≤ 6 months | 176 (30.3%) | 201 (30.1%) | **< 0.001** |
| Age > 6 months | 176 (30.3%) | 85 (12.7%) | **< 0.001** |
| Prematurity | 94 (16.2%) | 107 (16.0%) | 1.000 |

| | | | |
|---|---|---|---|
| Other risk factors* | 131 (22.6%) | 127 (19.0%) | 0.141 |
| Severity_mild° | 388 (66.9%) | 331 (49.6%) | **< 0.001** |
| Severity_moderate° | 184 (31.7%) | 304 (45.6%) | **< 0.001** |
| Severity_severe° | 8 (1.4%) | 32 (4.8%) | **< 0.001** |
| Fever | 81 (14.1%) | 71 (10.7%) | 0.084 |
| CXR | 350 (60.3%) | 529 (79.3%) | **< 0.001** |
| Bronchopneumonia | 126 (21.7%) | 245 (36.7%) | **< 0.001** |
| **Salbutamol aerosols, n (%)** | **1218 (38.1%)** | **1983 (61.9%)** | |
| PEDd | 392 (32.2%) | 1248 (62.9%) | **< 0.001** |
| SSO | 56 (4.6%) | 258 (13%) | **< 0.001** |
| Hospitalization | 770 (63.2%) | 477 (24.1%) | **< 0.001** |
| Age ≤ 3 months | 634 (52.1%) | 151 (7.6%) | **< 0.001** |
| Age ≤ 6 months | 289 (23.7%) | 609 (30.7%) | **< 0.001** |
| Age > 6 months | 295 (24.2%) | 1223 (61.7%) | **< 0.001** |
| Prematurity | 121 (9.9%) | 117 (5.9%) | **< 0.001** |
| Other risk factors* | 149 (12.2%) | 152 (7.7%) | **< 0.001** |
| Severity_mild° | 915 (75.1%) | 1602 (80.8%) | **< 0.001** |
| Severity_moderate° | 281 (23.1%) | 359 (18.1%) | **< 0.001** |
| Severity_severe° | 22 (1.8%) | 22 (1.1%) | **< 0.001** |
| Fever | 153 (12.6%) | 227 (11.6%) | 0.406 |
| CXR | 684 (56.2%) | 846 (42.7%) | **< 0.001** |
| Bronchopneumonia | 222 (18.2%) | 304 (15.3%) | **0.036** |
| **CXR, n (%)** | **1671 (52.2%)** | **1530 (47.8%)** | |
| PEDd | 1143 (68.4%) | 497 (32.5%) | **< 0.001** |
| SSO | 160 (9.6%) | 154 (10.1%) | **< 0.001** |
| Hospitalization | 368 (22.0%) | 879 (57.5%) | **< 0.001** |
| Age ≤ 3 months | 321(19.2%) | 464 (30.3%) | **< 0.001** |
| Age ≤ 6 months | 456 (27.3%) | 442 (28.9%) | **< 0.001** |
| Age > 6 months | 894 (53.5%) | 624 (40.8%) | **< 0.001** |
| Prematurity | 66 (3.9%) | 172 (11.2%) | **< 0.001** |
| Other risk factors* | 98 (5.9%) | 203 (13.3%) | **< 0.001** |
| Severity_mild° | 1427 (85.4%) | 1090 (71.2%) | **< 0.001** |
| Severity_moderate° | 237 (14.2%) | 403 (26.3%) | **< 0.001** |
| Severity_severe° | 7 (0.4%) | 37 (2.4%) | **< 0.001** |
| Fever | 163 (9.9%) | 217 (14.3%) | **< 0.001** |

Results are reported as total number, percentage and p-value (statistically significant if < 0.05). Significant differences for p-values are indicated in bold. PEDd: discharged from the Pediatric Emergency Department, SSO: short-stay observation unit; CXR: chest X-ray. *Other risk factors include: previous episodes of apnea, history of chronic lung or congenital heart disease, immunodeficiency, severe neurological or muscular disease; ° according to the Italian inter-society consensus document on bronchiolitis [11].
